# Green synthesis of silver and gold-doped zinc oxide nanocomposite with propolis extract for enhanced anticancer activity

**DOI:** 10.1038/s41598-024-71758-9

**Published:** 2024-09-18

**Authors:** Abdallah S. Abdelsattar, Aghapy Yermans Yakoup, Azza G. Kamel, Ayman El-Shibiny

**Affiliations:** 1 Center for Microbiology and Phage Therapy, Zewail City of Science and Technology, Giza, 12578 Egypt; 2 Faculty of Environmental Agricultural Sciences, Arish University, Arish, 45511 Egypt

**Keywords:** Anticancer, Nanocomposite, Silver nanoparticle, Gold nanoparticle, Zinc oxide nanoparticle, Biotechnology, Nanoscience and technology

## Abstract

Metal and metal oxide nanocomposites have unique properties and are promising for antibacterial and anticancer applications. In this work, we aimed to highlight the relationship between the biosynthesis ways of silver and gold-doped zinc oxide nanocomposites and their functions as anticancer on cell lines (MCF-7 and HepG2). The propolis was used to biosynthesize four different nanoparticles with the same components, including zinc, gold and silver. The nanocomposites were characterized using various techniques, including ultraviolet–visible spectroscopy (UV–Vis), scanning electron microscopy (SEM), transmission electron microscopy (TEM), Energy Dispersive X-ray analysis (EDX) and cytotoxicity assays. The result of this study showed that formed nanocomposites have a similar level of Zn, Au, and Ag, ranging from 23–34%, 2–6%, and 2–3%, respectively. In addition, adding the components simultaneously produces the fastest color change, and the fabricated nanoparticles have spherical shapes with different layers. In addition, the prepared nanoparticles influenced the cell viability of the cancer cell lines, with the most effective one when Zn, Au, and Ag were added spontaneously to form a nanocomposite called (All) with IC_50_ of 24.5 µg/mL for MCF7 cells and 29.1 µg/mL for HepG2 cells. Thus, the study illustrates that the preparation of nanocomposite generated through green synthesis with different methods significantly affects the structure and function and may improve the synthesis of nanocomposite to be developed into an efficacious therapeutic agent for cancers. In addition, this study opens the door toward a novel track in the field of nanocomposites as it links the synthesis with structure and function. Further anti-cancer properties, as well as animal testing are needed for those nanocomposites.

## Introduction

Throughout decades, various techniques have developed to boost the quality of life in different life aspects. In recent years, the most used technique is nanotechnology, which has been developed in numerous fields such as agricultural^1^ and biological fields^2^. Meanwhile, it is used in science and engineering to produce and control materials on small scales, such as the molecular and atomic scales^3,4^. Nanomaterials are the materials converted from their size in the normal scale to the nanoscale, between 1 and 100 nm^5,6^. Accordingly, materials in the nanoscale, nanoparticles (NPs), are shown to have different gained characteristics, chemically and physically^7,8^. Notably, these new characteristics for NPs have resulted in using them in aspects other than their normal use^9^. For instance, NPs are used in the biomedical field to manufacture different drug delivery systems^10^, antimicrobial agents^11,12^, and anticancer agents^13^.

In 1857, various metallic ions were used, for the first time, in synthesizing NPs to be used in the reduction process in industry^14,15^. Since then, the main three metallic ions that were used in the reduction process were gold (Au), silver (Ag), and zinc (Zn)^16,17^. Later, other applications were discovered for using these metallic ions in synthesizing NPs^18,19^. First, gold NPs (Au-NPs) were used in treating and tackling different tumors and cancer cells^20,21^. Second, silver NPs (Ag-NPs) were used in cosmetics^22,23^ and antioxidant agents^24,25^. Third, zinc NPs (Zn-NPs) were used as antibacterial agents^26^ and in gas sensors in agricultural fields^27^. Nowadays, other metallic ions can be used in synthesizing NPs like platinum (Pt), iron (Fe), and copper (Cu)^28,29^. The use of combinations among more than one metallic provides a higher performance and with lower concentration^30^. The use of zinc, gold, and silver as a combination had a promising activity against cancer cell lines^31^.

No sooner, distinct methods were established to synthesize different NPs like chemical, physical, biochemical and biosynthesis methods^32,33^. Although these methods have been used for years, the biosynthesis method has proven to have a better effect, less toxicity^34^, and higher yield^35,36^. Besides, biosynthesized NPs are easy to synthesize^37^, biodegradable^38^, and biocompatible^39^. For the biosynthesis of NPs, many natural components can be used, such as plants, algae, fungi, and bacteria^40–43^. Propolis is one of the natural components that could be used to biosynthesize NPs^44^. Whereas it is used as a resinous mixture^45^, which improves the effectiveness of the NPs^46^. Propolis is considered to be a coating agent used for metallic and metallic oxide nanoparticles as it contains many vital components such as waxes, essential oils, polyphenolic, aromatic acids, phenolic acid esters, flavonoids, and terpenoid components^47–49^. Thus, it has specific characteristics to be used in treatments such as antimicrobial^50^, anti-inflammatory^51^, antioxidant^52^, and antitumor agents^53,54^.

Recently, several forms and combinations between these metals have been triggered to enhance the effect of the biosynthesized NPs^55,56^. Eventually, these forms can be metals, metal oxides, and metal dioxides that might differ in the arrangement of the core and shell molecules and the polymer coating of the prepared NPs^57,58^. In 2017, Rossi et al. illustrated that combining NPs with polymers can improve the electrical and chemical properties of the metallic NPs^59^. Currently, studies demonstrate that combining Ag-NPs and Cu-NPs has better results in inhibiting bacterial cells than using each independently^60,61^. Furthermore, combining Zn-NPs and Ag-NPs was shown to have a stronger antibacterial effect on both Gram-positive and Gram-negative bacterial strains than using each of them unaccompanied^62–64^. However, these combinations were suggested to give this distinct activity due to their ability to trigger the formation of large amounts of reactive oxygen species, which would lead to DNA damage and cell injury if the cell was either a cancer cell or a bacterial cell^65–67^. Additionally, several studies have integrated the use of propolis extract as a reducing agent in preparing the metal nanoparticles, such as ZnO–propolis composite, which showed anticancer activity against several cell lines^68^. Moreover, our previous work using Au@AgNPs synthesized with propolis showed antibacterial and anticancer activity^69^. Therefore, further applications are suggested using different combinations from NPs^70,71^.

As mentioned, these combinations might be a new approach used in different life applications^72,73^. Consequently, more studies are needed to study the effect of different combinations and the reason for their additional distinctive function in these applications. In this study, we prepared four combinations that are made up of the same three metallic ions, Au, Ag, and Zn, but they are prepared with different orientations in the biosynthesis process. We aim to detect the differences between these components when they were prepared with different orientations. Furthermore, further studies are still needed to confirm the results of the prepared solutions and do further analysis.

## Materials and methods

### Preparation of *propolis*

The source of the propolis was gifted by the faculty of Environmental Agriculture Sciences, Arish University, to be prepared and used. The sample was prepared using the heated ethanol extraction method with modifications. Briefly, 0.4 g of the propolis was added to 10 mL of HPLC-grade ethanol 80% and incubated at 60 °C for 2 h, then 80 °C for 2 h. After the incubation, the sample was centrifuged for 15 min at 4 °C at 4000 xg. Then, the supernatant was filtered by a syringe filter. Finally, 1 mL of the resulting solution was evaporated to calculate the yield.

### Biosynthesis of nanoparticles

For biosynthesizing the four different NPs, a previous method^31,74^ was followed with modifications: AgNO_3_ of concentration 1 mM was mixed with HAuCl_4_ of concentration 1 mM and Zn(CH₃CO₂)₂ of concentration 100 mM. All these components were mixed in deionized water that contained 2% of the propolis extract. These steps were repeated four different times where the components were added in different arrangements, AgNO_3_, HAuCl_4,_ then Zn(CH₃CO₂)₂ to prepare “Ag-Au-ZnO-NPs,” or HAuCl_4_, Zn(CH₃CO₂)₂ the n AgNO_3_ to biosynthesize “Au-Zn-AgNPs,” or Zn(CH₃CO₂)₂, HAuCl_4_ then AgNO_3_ for forming “Zn-Au-AgNPs.” The time between adding each precursor in each nanocomposite was one hour. In the fourth nanocomposite, all the precursors were added simultaneously from the beginning to biosynthesize “All”. The solutions were left for 4 h at 85 °C with continuous stirring and in the presence of visible light.

### Characterization of nanoparticles

Various techniques were used to characterize the biosynthesized nanocomposites according to the literature^75^ with modifications. Briefly, UV-V spectroscopy (Jenway 7200, Jenway, UK) was used to determine the spectrum range between 340 and 800 nm, where 1000 µL of each compound was withdrawn and measured at the time points (60, 150 and 240 min) . The deionized water with 2% propolis was used as a control. Furthermore, the TEM (JEOL 1230, Japan) was used to determine their size and morphology, and the SEM to evaluate the topological surface and shape. EDX was used to determine the components and percentage of each biosynthesized nanoparticle. Finally, Zeta-potential Nano ZS (Malvern, UK) was used to measure the surface charge of the nanoparticles at 25 °C and voltage between -100 mV and 100 mV.

### Anticancer activity

The anticancer activity was done for the four nanoparticles on MCF-7 and HepG2 cell lines as described before^76^ with some modification. This was done as follows: in 96-well plates, 5000 cells/ well for both cell lines were cultured with DMEM medium supplemented with 10% fetal bovine serum and 0.5% penicillin–streptomycin, with a seeding density of 5000 cells/well. Cells grew to a monolayer for about 24 h at 37 °C/ 5% CO_2_ incubator. Then, the medium was replaced with a serial concentration of each nanoparticle individually (10, 20, 50, 100, 200, 500, and 1000 μg/mL) beside the negative control without treatment and incubated for 72 h at 37 °C/ 5% CO_2_ incubator.

Then, the cell viability was assessed using two different methods, MTT and Trypan blue assay. The MTT assay was conducted as the standard protocol^77^ by adding MTT reagent {10 μL of the stock concentration (5 mg/ml)/well} and incubating for 4 h at 37 °C/ 5% CO_2_ incubator. The medium was discarded, and 150 μL of DMSO was added to solubilize the formazan crystal, incubated for 20 min, and OD was measured at 570 nm. For the trypan blue assay, the cells were detached by adding 40 μL of trypsin in each well and incubated at 37 °C for 5 min, followed by adding 60 μL of complete DMEM medium. The number of viable cells was counted using a trypan blue reagent under an inverted microscope (Optika IM-3, Italy).

### Statistical analysis

The cytotoxicity experiments were conducted three times to ensure accuracy (as technical replications), and the results were confirmed through two separate experiments (as biological replications). Graphs with standard deviation (SD) error bars were created using Prism GraphPad version 5.01 for Windows software. The significance of the cytotoxicity was determined using a one-way ANOVA test.

## Results

### Color change and UV-V spectroscopy

The color change during the multi-step preparation was used to predict the formation of different nanocomposites. The water used is colorless, and after adding ethanolic propolis extract (2% v:v) a faint yellowish color starts to appear. After adding the first component in Zn-Au-AgNPs, Ag-Au-ZnO-NPs, or Au-Zn-AgNPs, a minor change in color was noticed. However, after adding all of the components in the case of a nanocomposite called “All,” the faint yellowish color changed to a strong dark color during the first hour, and no significant difference occurred in color during the time of formation. For Ag-Au-ZnO-NPs, after adding the second component, the color moved to a dark gray color, and the color became stronger after adding the third component. Finally, in Au-Zn-AgNPs and Zn-Au-AgNPs, the color dramatically changed from dark to dark gray after adding the second and third components, respectively (Figure S1).

**Fig. 1:**
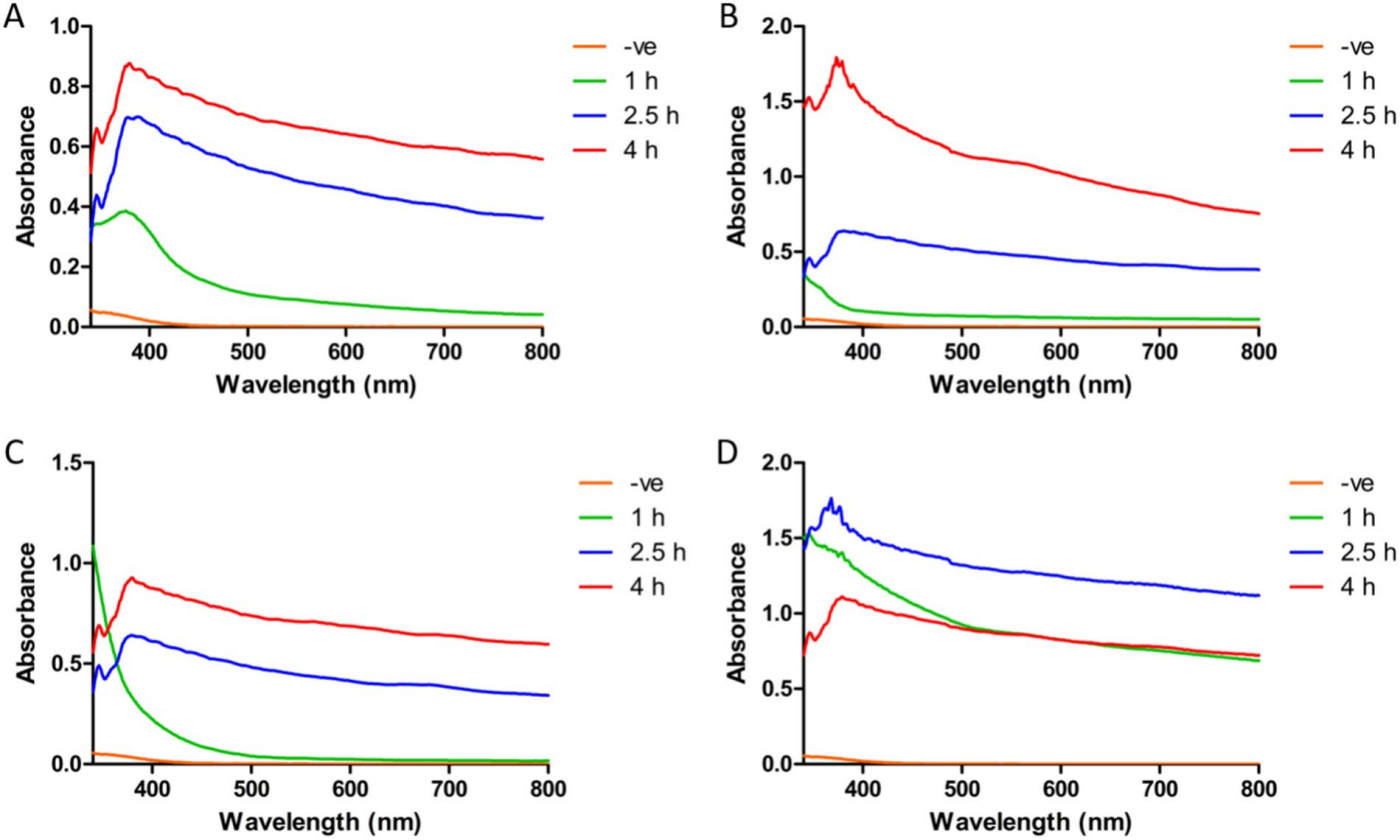
Shows the change in the wavelength absorption that occurs during the formation of (**A**) Ag-Au-ZnO-NPs, (**B**) Au-Zn-AgNPs, (**C**) Zn-Au-AgNPs, and (**D**) All.

The results show the changes in wavelength absorption between 340 and 800 nm for the different nanocomposites at different time points during their formation (Fig. 1). The chosen time points were after an hour of adding the first component, 90 min of adding two components, and 90 min after adding the three. Ag-Au-ZnO-NPs after the first measurement, there is a peak at wavelength ~ 390 nm with OD of ~ 0.4, representing the formation of AgNPs and increased with adding different components unlike “All” which has the highest readings after 2.5 h with no increase during the time. For Au-Zn-AgNPs and Zn-Au-AgNPs, the OD values for the peaks at ~ 390 increased by adding different components.

### TEM and SEM analyses

**Fig. 2:**
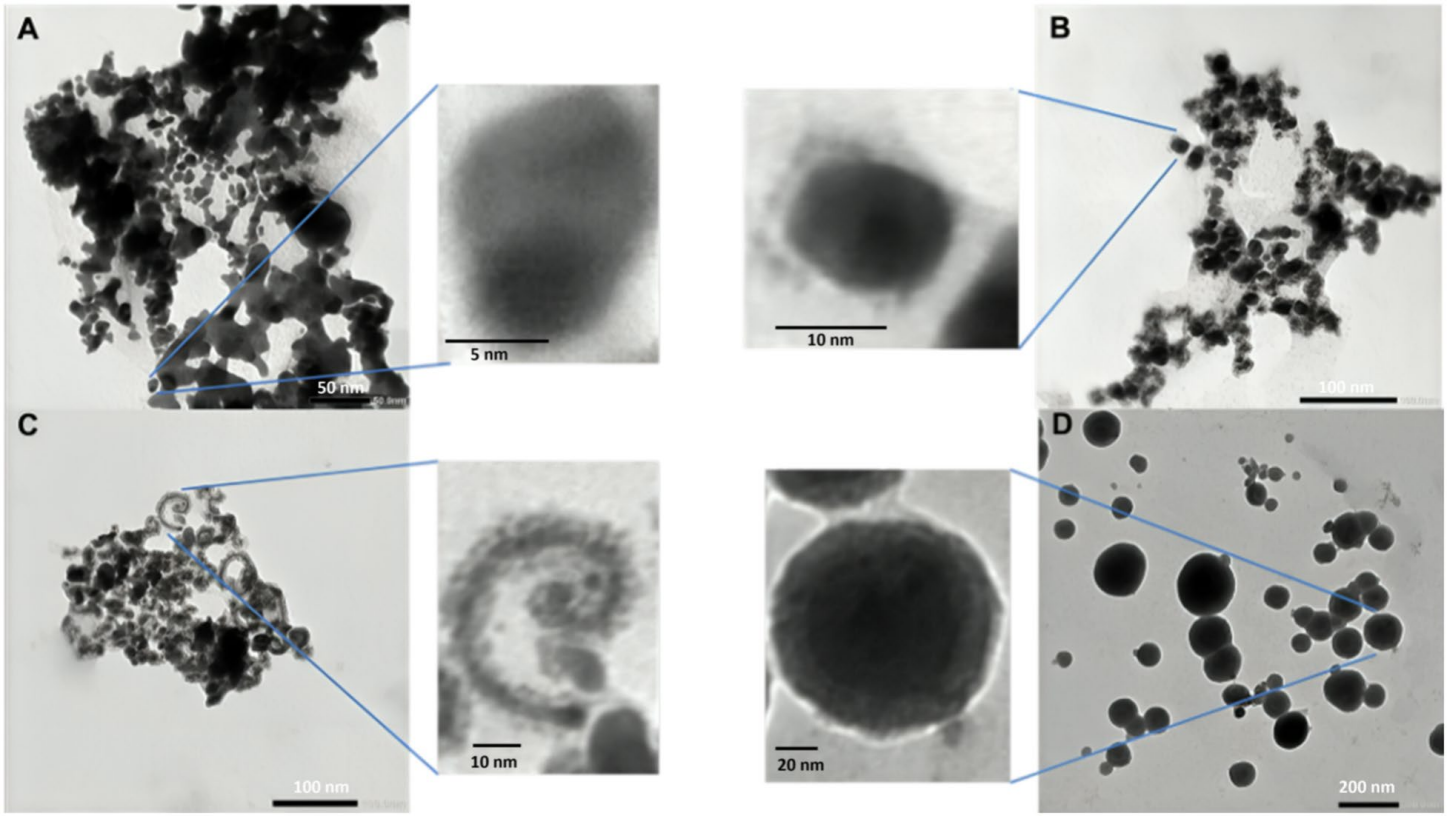
Illustrates the different shapes of nanocomposites under TEM. (**A**) Ag-Au-ZnO-NPs with a scale bar of 50nm, (**B**) Au-Zn-AgNPs with a scale bar of 100nm, (**C**) Zn-Au-AgNPs with a scale bar of 100nm, and (**D**) All with a scale bar of 200nm.

**Fig. 3:**
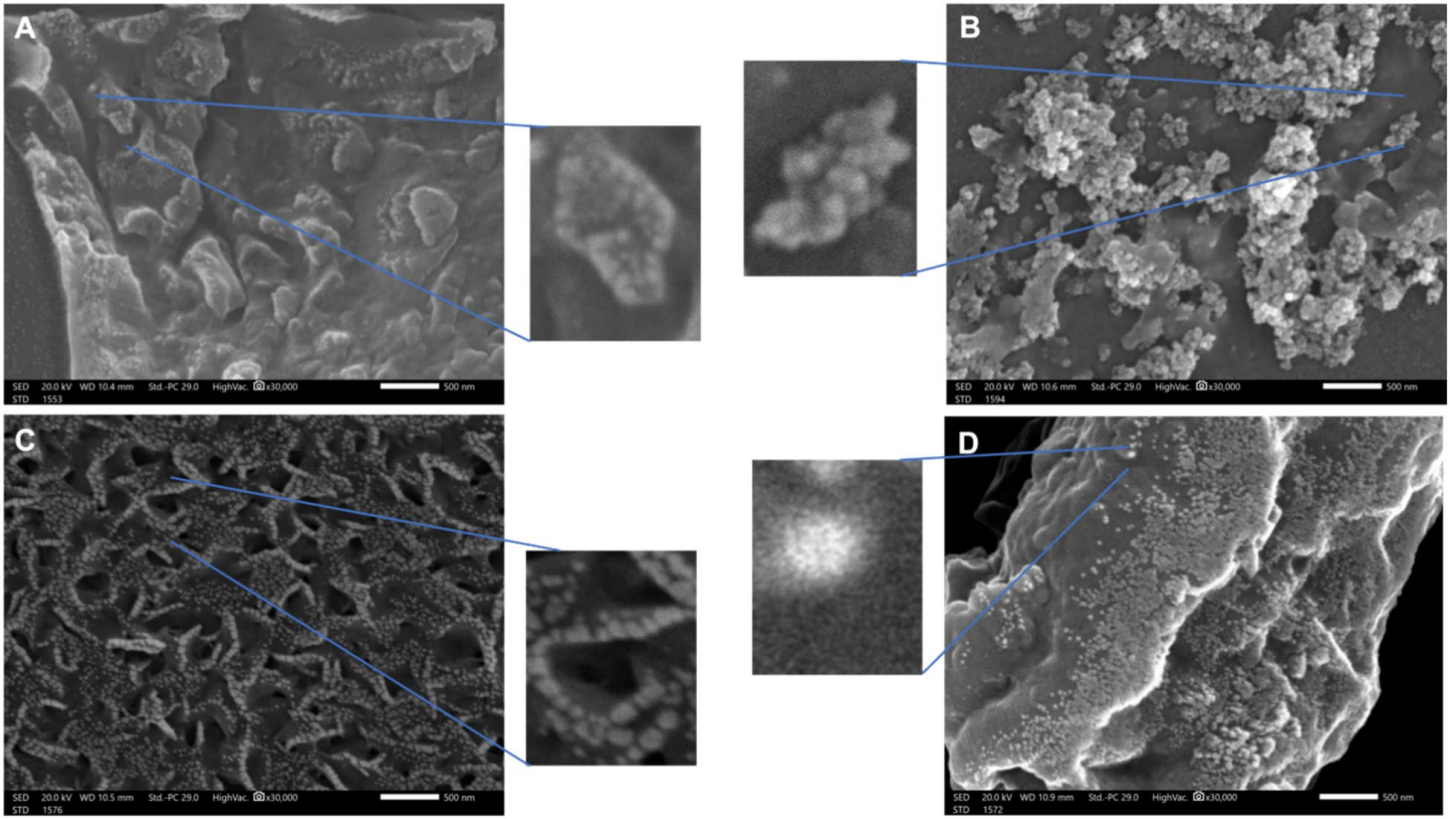
Shows the different shapes of nanocomposites under SEM. (**A**) Ag-Au-ZnO-NPs, (**B**) Au-Zn-AgNPs, (**C**) Zn-Au-AgNPs, and (**D**) “All”. The scale bar is 500nm.

**Fig. 4:**
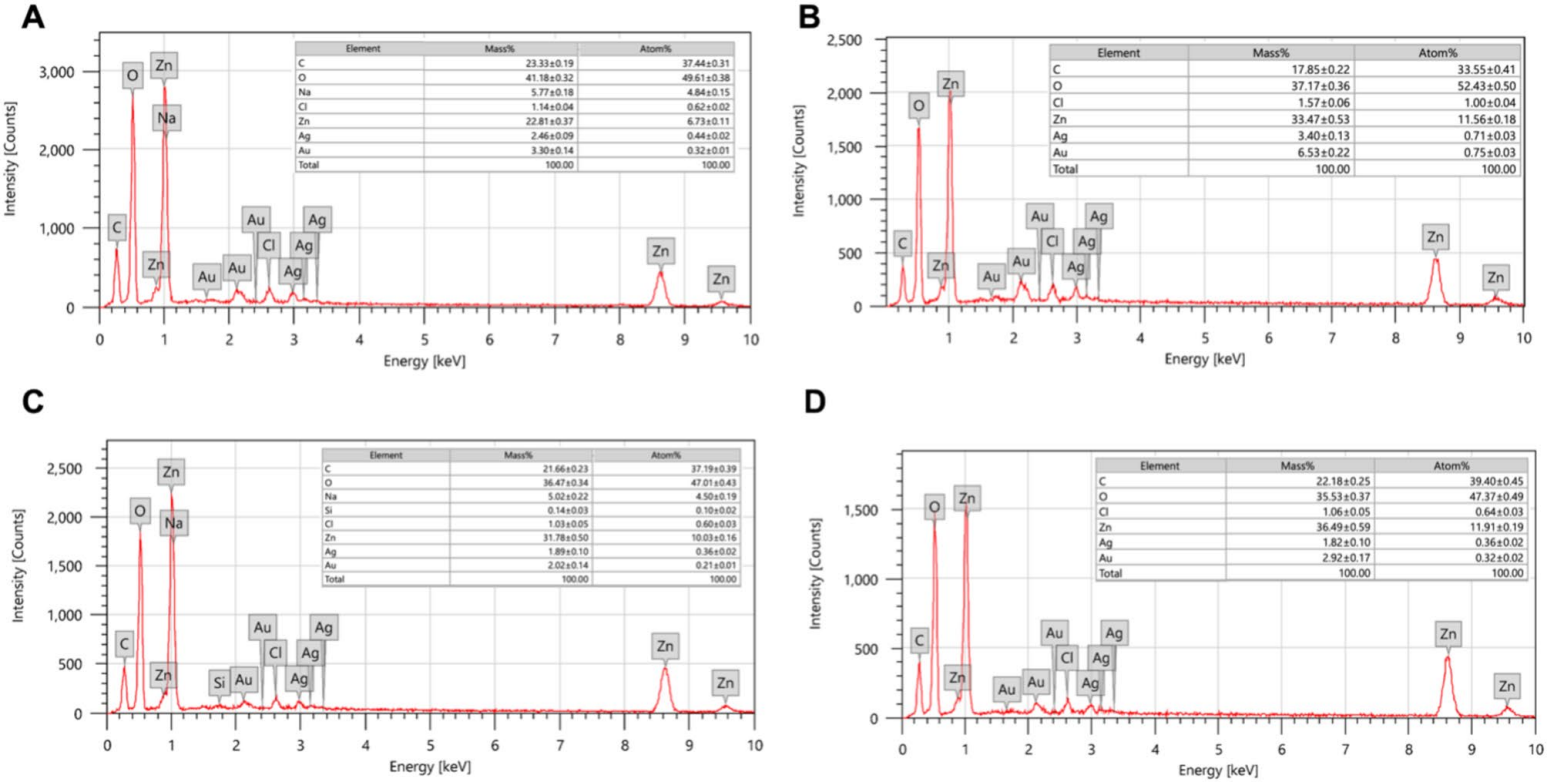
Shows the EDX analysis for the different nanocomposites. (**A**) Ag-Au-ZnO-NPs, (**B**) Au-Zn-AgNPs, (**C**) Zn-Au-AgNPs, and (**D**) “All”.

For more confirmation of the formulation of nanocomposites in the size of the nanostructure structure and to compare the differences in structure and shape of nanocomposite samples, both SEM and TEM were used (Figs. 2 and 3). For Ag-Au-ZnO-NPs, the SEM and TEM micrographs in Figs. 3A and 4A displayed irregular shapes with differences in color intensities. For Au-Zn-AgNPs, the SEM and TEM figures in Figs. 3B and 4B illustrated that the nanoparticles have irregular shapes similar to Ag-Au-ZnO-NPs. However, it was noted that small particles surrounded the nanoparticles. For Zn-Au-AgNPs in Figs. 3A and 4A, the shape was spiral and surrounded by small particles. Finally, the shapes for nanoparticles in “All” have the most regular spherical shape and are more organized than the others. It was noticed that most nanoparticles in “All” are larger than the others. In addition, the aggregation in “All” is minimal compared to the formulations from the same component but in different fabrication orders. Additionally, different layers inside each sphere represent a core–shell model of nanoparticle formation. The results from SEM are consistent with those of TEM.

### EDX and Zeta-potential analyses

The elemental analyses of different nanoparticles were measured using the energy dispersive X-ray analysis (EDX). Figure 4A illustrates the Ag-Au-ZnO-NPs with a zinc mass of 22.8%, 2.5% silver, and 3.3% gold, but most of the mass is oxygen and carbon from the organic source (propolis extract). On the other hand, Fig. 4B shows a high level of gold at 6.5%, a high level of silver at 3.4%, 33.4% of zinc, and a low level of carbon at 17.8%. In addition, the results from EDX show a similar percentage of elements in both preparations (Zn-Au-AgNPs and “All”), as shown in Fig. 4C, D. In Zn-Au-AgNPs and “All”, the carbon percentage is 21.6–22.1%, Oxygen percentage is 35.5–36.4%, Zinc percentage is 31.7–36.4%, the silver percentage is 1.8% and the gold percentage is 2–2.9%.

**Fig. 5:**
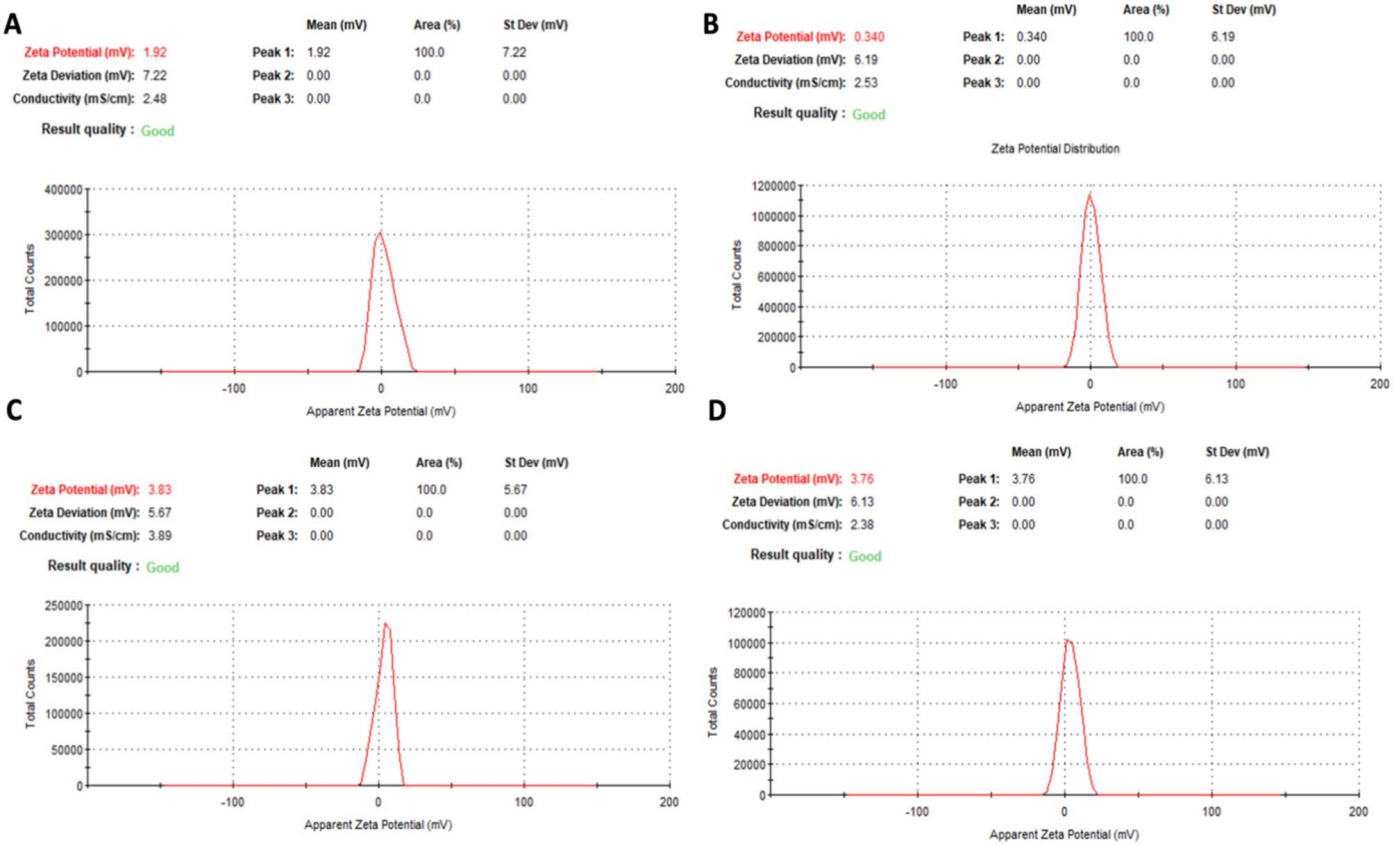
Shows the zeta-potential results for the different nanocomposites. (**A**) Ag-Au-ZnO-NPs, (**B**) Au-Zn-AgNPs, (**C**) Zn-Au-AgNPs, and (**D**) All.

The measurements of zeta potential value illustrate the combination of zinc, silver, gold, and propolis extract can interact and impart a positive charge to the surface of the nanoparticles. However, the analysis of the zeta-potential results reveals that the prepared nanocomposites have similar zeta potential ranging from + 0.4 to + 3.8 mV, as in Fig. 5. The low values of nanoparticles could result from using different metallic materials to form the nanocomposite, which can affect the stability, aggregation, and agglomeration states of the nanocomposite. These results are consistent with TEM images, as the aggregates can be noticed in Fig. 2.

### The anticancer activity

**Fig. 6:**
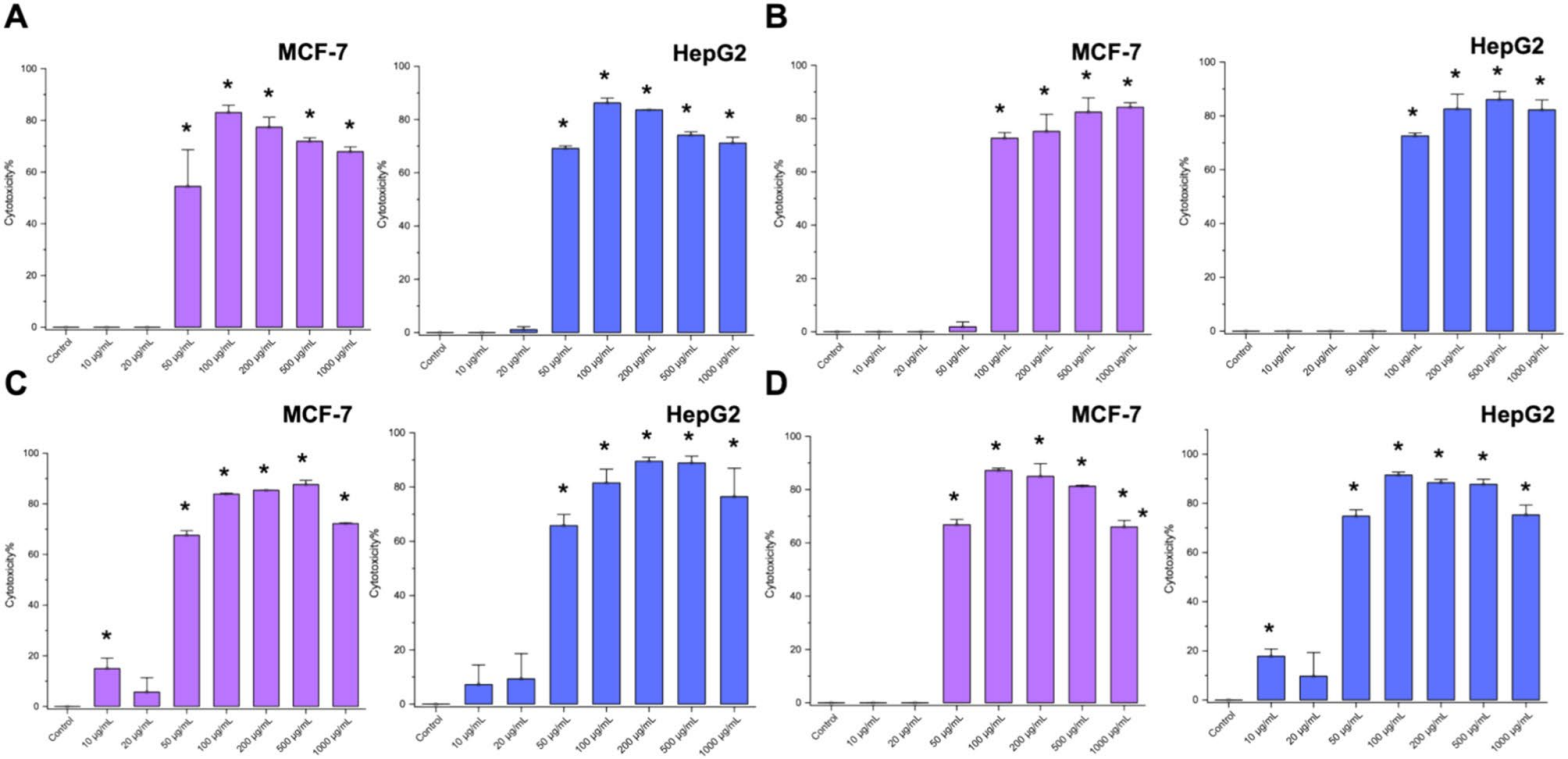
MTT results for the cytotoxicity effect of different nanoparticles (**A**) Ag-Au-ZnO-NPs, (**B**) Au-Zn-AgNPs, (**C**) Zn-Au-AgNPs, and (**D**) All against two different cell lines (MCF-7 and HepG2). * indicates *P*value < 0.05.

**Fig. 7:**
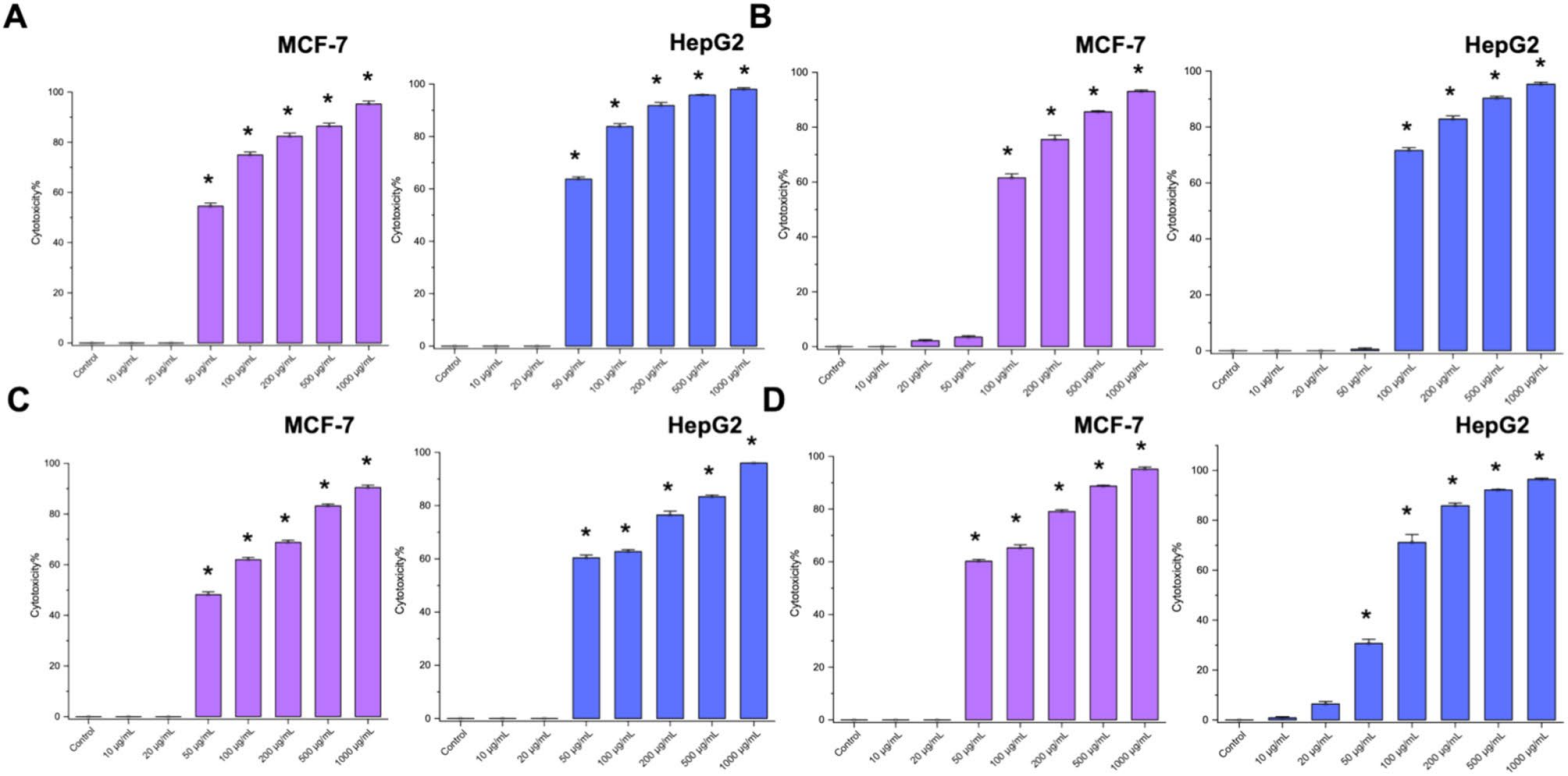
Trypan blue results for the cytotoxicity effect of different nanoparticles (**A**) Ag-Au-ZnO-NPs, (**B**) Au-Zn-AgNPs, (**C**) Zn-Au-AgNPs, and (**D**) All against two different cell lines (MCF-7 and HepG2). * indicates *P*value < 0.05.

The potential anticancer activity was evaluated by two methods (MTT and Trypan blue) on both types of cells (MCF-7 and HepG2) to investigate the growth inhibitory effect of the four nanoparticles. The four nanoparticles have shown a significant reduction in the cell viability percentage in a concentration-dependent mode (Figs. 6 and 7). However, there was a difference in the inhibitory effect among the four nanoparticles. According to the MTT results, the most effective one is All nanoparticles with IC_50_ of 24.5 µg/mL for MCF7 cells and IC_50_ of 29.1 µg/mL for HepG2 cells (Fig. 6D). The other nanoparticles have a variety of inhibitory effects on both cell lines, whereas Zn-Au-AgNPs (Fig. 6C) is the second most effective one on MCF7 with IC_50_ of 39.3 µg/mL compared to Ag-Au-ZnO-NPs (Fig. 6A) and Au-Zn-AgNPs (Fig. 6B) with IC_50_ of 40.4 µg/mL and 65 µg/mL, sequentially. For HepG2 cells, Ag-Au-ZnO-NPs (Figure &A) were the second most inhibitory effective nanoparticles with IC_50_ 31.6, followed by Zn-Au-AgNPs (IC_50_ 34.2 µg/mL) (Fig. 6C) and Au-Zn-AgNPs (IC_50_ 87.36 µg/mL) (Fig. 6B), sequentially. These results indicate that the sequence of adding the metals in nanoparticle preparation affects its efficiency and cytotoxicity effect on both types of cells.

The trypan blue results were conducted to confirm the cell viability compared to the MTT assay, regardless of the high concentration of nanoparticles at (500 and 1000 µg/mL), which makes it more precise (Fig. 7). Microscopic images confirm the results and represent the cell destructions and damages throughout the concentration gradients in both MCF-7 (Figure S2) and HepG2 (Figure S3). The results of trypan blue depicted the lower cytotoxicity for Au-Zn-AgNPs than the rest of the nanoparticles, which are still consistent with the results from the MTT assay.

## Discussion

Nanoparticles have several applications in biomolecules and drug delivery, and that is because of their unique structure^78^. In addition, their uptake by phagocytic cells was proved, which enhanced their function as carriers for several drugs and antibiotics^79^. Understanding the properties of nanoparticles, such as size and morphology, was essential in determining material properties such as nanoparticle crystals and their structure defect^80^, optical absorption cross-Sect.^81^ , and surface plasmon resonance^82^. Those properties, including drug delivery, are highly significant for biomedicine applications^79^. Mineral nanoparticles are significantly different according to their bulk analogs. The functions of size and formation conditions depend on the chemistry, surface structure, and degree of order^83^. One of those nanoparticles is ZnO NP, as their toxicity effect can be changed depending on their properties, such as morphology and size^84^. One study has shown the effect of different stabilizing agents through the formulation process of ZnO NPs as antibacterial against *E. coli* and *S. aureus*^85^. It has proven that superior antibacterial activity was observed when using the spherical particles synthesized with polyvinyl alcohol (PVA) compared with the hexagonal and ellipsis shapes because they presented a larger surface area and smaller size^85^. With Ag NPs, the stability of nanoparticle formation in several aqueous environments has a high effect on their activity because it affects the size of NPs, morphology, and surface-to-volume ratio, and all of those significantly affect the activity^86^. A study was conducted by Kvítek et al. (2008) to evaluate the use of different surfactants (ionic, nonionic, and polymers) in modifying the AgNPs’ surface. It has shown that ionic surfactant (sodium dodecyl sulfate-SDS) exhibited the highest stability to electrostatic repulsion and steric effect, leading to the most increased antibacterial activity compared to nonionic and polymers surfactants^87^. In addition, Au NPs have been proven to have various stabilities according to biosynthesis with several ligands to surface modification, affecting the structural properties and their activity against microorganisms and cancer cells^88^. This has been shown in a study when using plant extract (Brazilian Red Propolis) and its fractions, such as hexane, dichloromethane, and ethyl acetate. Accordingly, the size and morphology were influenced. The results show that dichloromethane and hexane fractions have increased the antibacterial activity, and the extract and dichloromethane have the highest cytotoxic effect on cancer cells^88^. This structure–chemistry relationship leads to the prediction of manufactured materials in a suitable manner for seeking better biological connections.

Metal nanoparticles have been widely used as an anticancer due to their ability to produce reactive oxygen species (ROS) in cellular compartments, eventually activating the apoptosis and necrosis death pathways^89^. Several studies have been done using metal nanoparticles such as gold, silver, and zinc against a wide range of cancer cell lines and proven to have anticancer activity using the metals in single or in combination with others. One study in which gold nanoparticles were used showed the anticancer activity on HepG2 and A549 (human lung alveolar epithelial) cell lines with a good result at 25 μg/mL for both cell lines^90^. Another work by Roshanak and others against MCF-7, HepG2 cancer cell lines in which they have used *B. citriodora* leaf extract for the synthesis of BCAu-NPs has proven the anticancer activity with IC_50_ of 116 and 108 µg in MCF-7 and HepG2 cancer cells, respectively^91^. Besides, nano-ZnO materials have been reported for their cytotoxicity effect on multiple cancer cell lines. In a study where the ZnFe_2_O_4_ nanoparticles were tested against the MCF-7 cell line, the cell viability significantly decreased by 44% after 24 h incubation with 100 µg/mL^92^. Another study has shown the cytotoxicity of ZnO NPs on MCF-7, HepG2, and A-549 cells at various concentrations (1–100 µg/mL), and the results showed a decrease in cell viability with increasing ZnO NP concentration^92^. Similarly, silver nanoparticles were widely used in biomedical research as an anticancer. A study was done by Lan Anh Thi Nguyen and others have used synthesized AgNPs with the aqueous extract of *C. fragrans* leaves as anticancer against several cancer lines, and they found a significant decrease in cell viability on both MCF-7 and HepG2 cancer cell lines with the IC_50_ values of 2.41 and 2.31 µg/mL, respectively^93^.

This work provides valuable insights into the consequences of preparing the nanocomposite with the same concentrations of the same material, including how this affects the structure and function. However, further studies are needed to understand the potential of these nanocomposites. The studies should include; (i) confirming the anticancer properties by conducting 2D and 3D analyses, as well as animal testing to track the alterations in biochemical parameters and tissue morphology in rats^94,95^; (ii) Conducting more characterization analysis, including X-ray Diffraction, thermogravimetric analysis, and Fourier-transform Infrared Spectroscopy are also needed^31,96^ in addition to the (iii) evaluation of other activities such as the antioxidant, metal detection, and degradation dyes^24,75^. Finally, (iv) expand the concept to include different combinations of materials.

## Conclusion

In summary, we presented different biosynthesis methods to produce nanocomposite consisting of zinc, gold, and silver using ethanolic propolis extract. The goal of this comparison is to relate the structure and function with the method preparation and to be a nucleus for future work on the same track. In this work, the green nanocomposites were characterized by tracking the color change during the time, UV–visible analysis, visualization using TEM and SEM, Zeta potential, and EDX. Moreover, the cancer cell lines were used to evaluate the cytotoxicity. It was found that zinc combined gold and silver as nanocomposite in “All” exhibited a superior anticancer activity over the other preparation methods. In addition, the formation of nanoparticles changes from one preparation to another in terms of the shape, size, potential anticancer activity, and time needed to be prepared. Finally, we concluded that adding the component in different ways can change the formation of nanoparticles in structural and functional matter. The potential anticancer activity opens the door to more research on enhancing the activity for using nanocomposites in future biomedical applications.

## Supplementary Information

Supplementary Information.

## Data Availability

Data is provided within the manuscript or supplementary information files.
