# Supplementary material for: Green synthesis of silver and gold-doped zinc oxide nanocomposite with propolis extract for enhanced anticancer activity
Source: Sci Rep. 2024 Sep 18;14:21763. doi: 10.1038/s41598-024-71758-9 (PMC11410827; doi:10.1038/s41598-024-71758-9)
Supplement: Supplementary file 1 — Supplementary Information. [file 41598_2024_71758_MOESM1_ESM.docx]

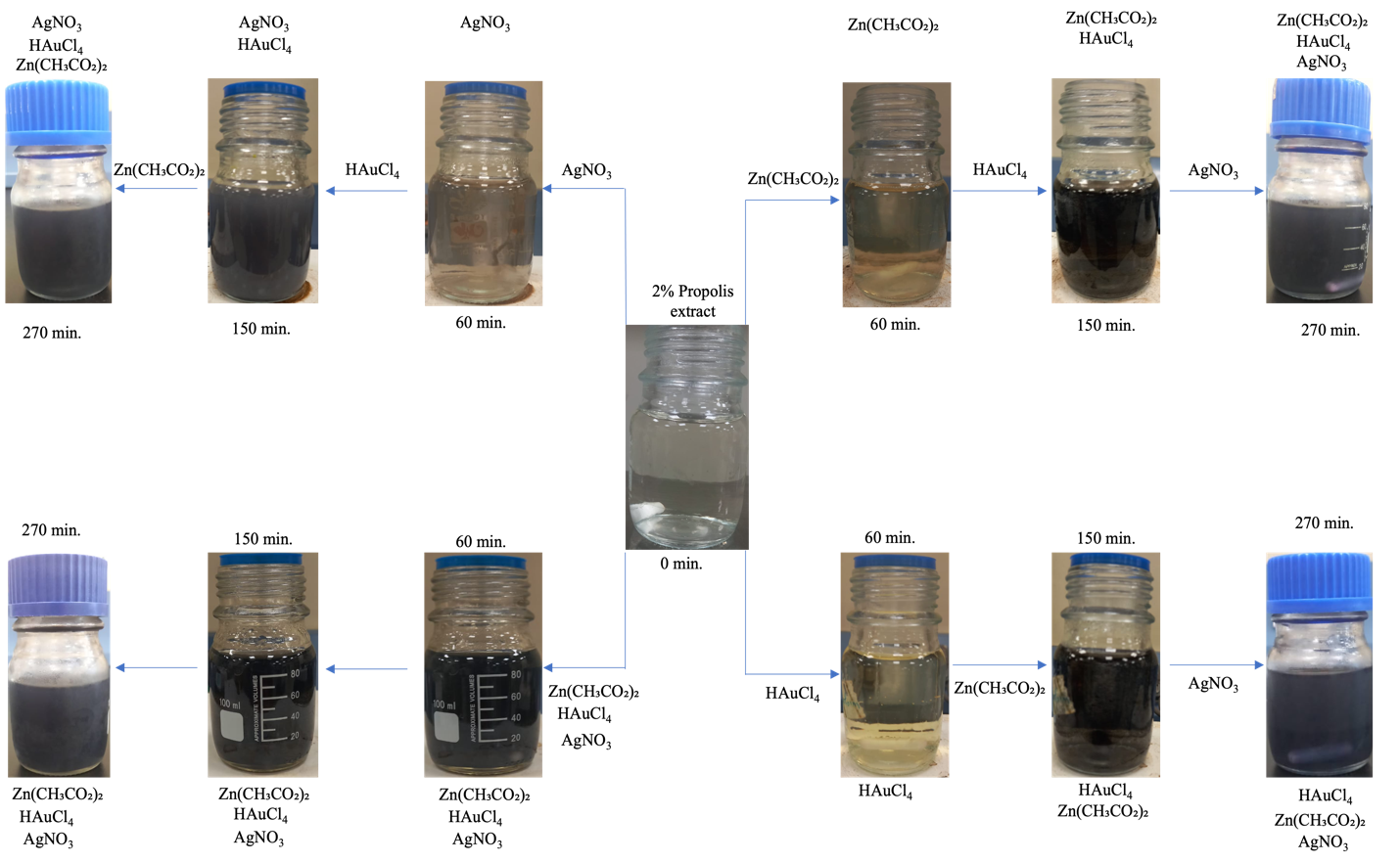


Figure S1: The change in color during the process of formation of different nanocomposites: the upper left for “Ag-Au-ZnO-NPs”, the upper right for “ZnO-Au-AgNPs”, the lower left for “All”, and the lower right for “Au-Zn-AgNPs”.


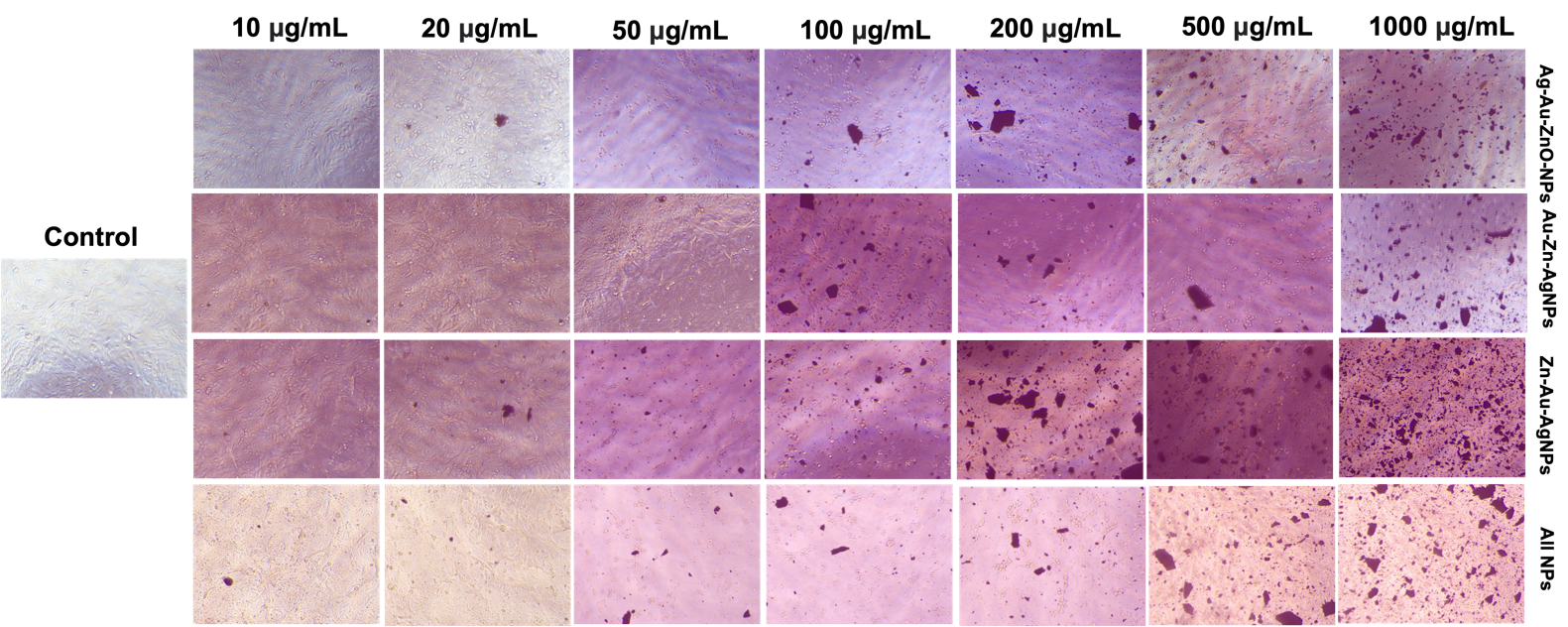


*Figure S2: Microscopic images represent the cytotoxicity of the synthesized nanoparticles on MCF-7 cell lines compared to the control.*

*
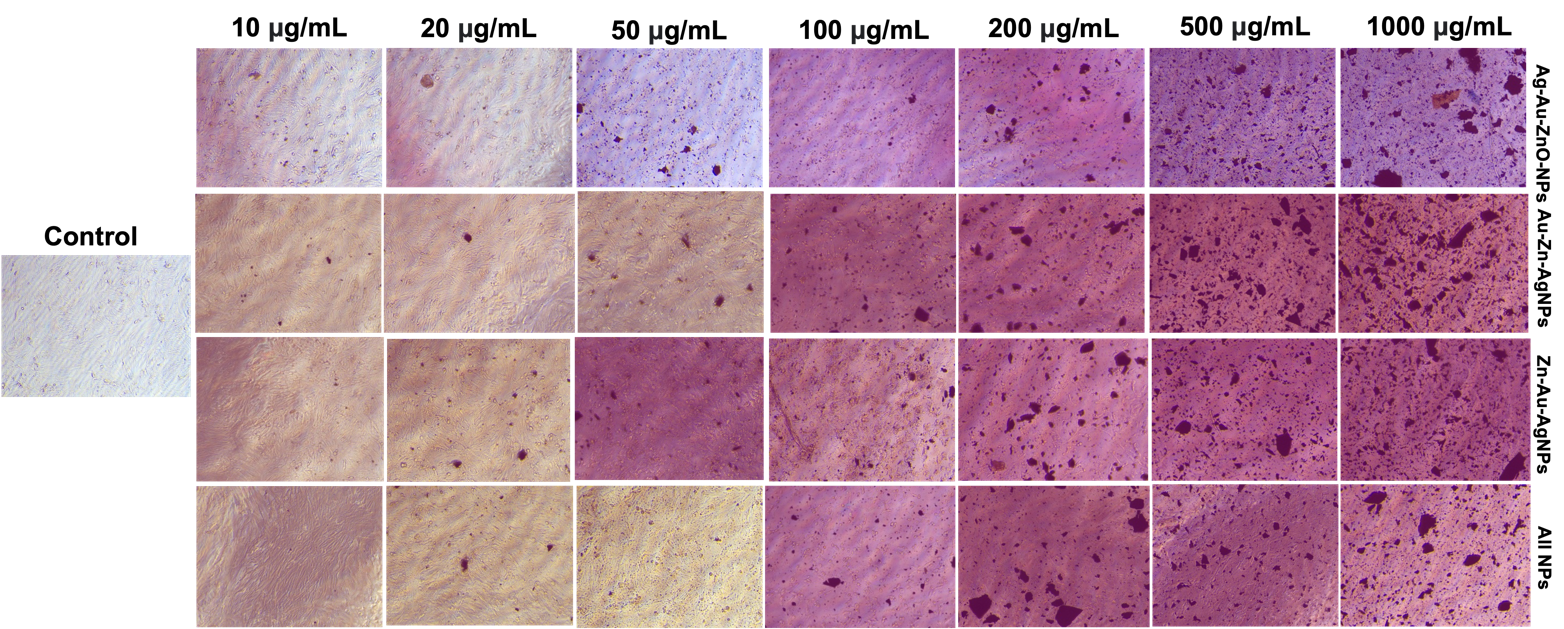
*

*Figure S3: Microscopic images represent the cytotoxicity of the synthesized nanoparticles on HepG2 cell lines compared to the control.*
